# Retraction: New insights into virulence mechanisms of rice pathogen *Acidovorax avenae* subsp. *avenae* strain RS-1 following exposure to ß-lactam antibiotics

**DOI:** 10.1038/srep47003

**Published:** 2018-07-16

**Authors:** Bin Li, Mengyu Ge, Yang Zhang, Li Wang, Muhammad Ibrahim, Yanli Wang, Guochang Sun, Gongyou Chen

Scientific Reports 6: Article number: 2224110.1038/srep22241; published online: 02 26 2016; updated: 07 16 2018

The authors are retracting this Article as there are duplications, and inappropriate manipulations, of images in the figures.

Images are duplicated between Figures 1b (Amo (+)/Pen (+)) and Figure 1d (WT/Δ*hcp*); and between Figures 1c (Pen (+)) and Figure 1e (Δ*hcp*).

Images are duplicated between the upper panel of Figure 2a (*rfp* (−)/None) and Figure 2b (WT/None).

In Figure 6c (Cytoplasmic proteins) the Δ*hcp* well is taken from a different plate to the other wells.

In Figure 7a: the Hcp/PKNT25 and PCH363/Lipo images are derived from the same original image; and the PCH363/VgrG-A and PCH363/PKNT25 images are derived from the same original image.

The pull-down assay reported in Figure 7b does not include input, output or negative control lanes and cannot be relied upon.

The authors apologize for these issues, which undermine full confidence in the integrity of the study.

All authors agree to the retraction.

